# Interactive effects of temperature, organic carbon, and pipe material on microbiota composition and *Legionella pneumophila* in hot water plumbing systems

**DOI:** 10.1186/s40168-017-0348-5

**Published:** 2017-10-04

**Authors:** Caitlin R. Proctor, Dongjuan Dai, Marc A. Edwards, Amy Pruden

**Affiliations:** 1 Via Department of Civil and Environmental Engineering, Virginia Tech, Blacksburg, Virginia, 24061 USA; 2 EAWAG, Swiss Federal Institute of Aquatic Science and Technology, Überlandstr 133, CH-8600 Duebendorf, Switzerland

**Keywords:** Hot water, Premise plumbing, *Legionella*, Opportunistic pathogen, Temperature control, Pipe material

## Abstract

**Background:**

Several biotic and abiotic factors have been reported to influence the proliferation of microbes, including *Legionella pneumophila*, in hot water premise plumbing systems, but their combined effects have not been systematically evaluated. Here, we utilize simulated household water heaters to examine the effects of stepwise increases in temperature (32–53 °C), pipe material (copper vs. cross-linked polyethylene (PEX)), and influent assimilable organic carbon (0–700 μg/L) on opportunistic pathogen gene copy numbers and the microbiota composition, as determined by quantitative polymerase chain reaction and 16S rRNA gene amplicon sequencing.

**Results:**

Temperature had an overarching influence on both the microbiota composition and *L. pneumophila* numbers. *L. pneumophila* peaked at 41 °C in the presence of PEX (﻿1.58 × 10^5^ gene copies/mL). At 53 °C, *L. pneumophila* was not detected. Several operational taxonomic units (OTUs) persisted across all conditions, accounting for 50% of the microbiota composition from 32 to 49 °C and 20% at 53 °C. Pipe material most strongly influenced microbiota composition at lower temperatures, driven by five to six OTUs enriched with each material. Copper pipes supported less *L. pneumophila* than PEX pipes (mean 2.5 log_10_ lower) at temperatures ≤ 41 °C, but showed no difference in total bacterial numbers. Differences between pipe materials diminished with elevated temperature, probably resulting from decreased release of copper ions. At temperatures ≤ 45 °C, influent assimilable organic carbon correlated well with total bacterial numbers, but not with *L. pneumophila* numbers. At 53 °C, PEX pipes leached organic carbon, reducing the importance of dosed organic carbon. *L. pneumophila* numbers correlated with a *Legionella* OTU and a *Methylophilus* OTU identified by amplicon sequencing.

**Conclusions:**

Temperature was the most effective factor for the control of *L. pneumophila*, while microbiota composition shifted with each stepwise temperature increase. While copper pipe may also help shape the microbiota composition and limit *L. pneumophila* proliferation, its benefits might be constrained at higher temperatures. Influent assimilable organic carbon affected total bacterial numbers, but had minimal influence on opportunistic pathogen gene numbers or microbiota composition. These findings provide guidance among multiple control measures for the growth of opportunistic pathogens in hot water plumbing and insight into the mediating role of microbial ecological factors.

**Electronic supplementary material:**

The online version of this article (10.1186/s40168-017-0348-5) contains supplementary material, which is available to authorized users.

## Background

Preventing proliferation of opportunistic pathogens (OPs) in building plumbing is an important step in delivery of safe drinking water to consumers. In particular, *Legionella pneumophila* is an OP that has become the leading cause of drinking-water-related disease in developed countries [1]. *L. pneumophila* is especially challenging to control because it establishes and grows as part of the native plumbing microbiota, even under oligotrophic conditions and in the presence of disinfectants. *L. pneumophila* can thrive in biofilms, partly owing to the protection provided by amoeba hosts, inside which they preferentially amplify [2]. Some amoebae occurring in drinking water, such as *Acanthaomeba* spp. and *Naegleria fowleri*, are also OPs [3–5]. In considering options for intentionally shaping the microbiota in building plumbing systems, temperature and pipe material are practical factors that can be directly controlled at the building level, while assimilable organic carbon (AOC) is influenced by both municipal water treatment and conditions in the building plumbing.

Elevating the water temperature > 60 °C can be highly effective for killing and inhibiting *L. pneumophila* and other OPs [6, 7]. However, the reality is that such “hot” temperatures are difficult to maintain throughout the system, resulting in “warm” water conditions that are highly conducive to pathogen growth in much of the building plumbing environment [8]. Water heaters pose a risk for promoting microbial illness when operated between 30 and 54 °C [9] or whenever the water coming out of the heaters is below 60 °C or fails to remain consistently above 55 °C throughout the premise plumbing [10]. Despite the well-established benefits of elevated temperatures for OP control, some guidelines for water heater operation recommend temperatures as low as 48 °C [11, 12] because of concerns about scalding, scaling, and energy conservation, with only some recommending higher temperatures [13–15]. Besides direct effects on pathogens, temperature is a fundamental selective force in shaping the broader microbiota of tap water [16].

Within a building, pipes and plumbing components are made from a vast array of materials, all with the potential to influence microbial regrowth [17]. The high surface area to volume ratio in the home compared to that of the main water distribution system can exacerbate microbial growth, while the higher temperatures encountered in hot water lines can mediate the influence of materials [17, 18]. Plastic pipes, especially cross-linked polyethylene (PEX), can release substantial amounts of organic carbon, stimulating growth of heterotrophic bacteria and amoebae [18–21]. Plastic pipes can also release phosphate, antioxidants, and complex non-biodegradable organics [19, 22, 23]. Copper, on the other hand, has antimicrobial properties and is commonly applied together with silver for on-site disinfection [24, 25]. However, when used as a pipe material, the disinfectant properties of copper can vary [26–29]. For example, in one model distribution system, copper pipe seemed to control *L. pneumophila* growth, but only for a limited time [21], while in a field study, positive correlations between copper fixtures of unknown age and *L. pneumophila* were noted [29]. Another study found no difference in *Legionella* spp. abundance associated with PEX versus copper in an office building, but did note that copper suppressed other potential OPs (i.e., *mycobacteria*), demonstrating that different OPs can vary in their associations with materials and that individual microbiota members can behave differently [16].

Limiting the levels of nutrients in treated and distributed water could enable water utilities to reduce risk of pathogen regrowth at the community-scale. AOC is a common target, with proposed limits of 10 μg/L [30] and 32 μg/L [31] in non-disinfected water or 100 μg/L in water carrying disinfectant residual [32]. However, it is important to also consider that AOC can be produced during distribution and in building plumbing, for example, via nitrifying and other autotrophic organisms [33] or through leaching from plastic pipes [34]. Moreover, expectations for AOC control have been based on studies of distribution system water under continuous-flow conditions, whereas premise plumbing operates under warm semi-stagnant conditions. A prior controlled study simulating the premise plumbing environment noted that total organic carbon level was not the master variable controlling proliferation of *L. pneumophila* or *Mycobacterium avium* [35].

Here, we employed simulated water heaters (SWH) to establish a fundamental understanding of interactive effects of temperature, pipe material, and AOC levels on the microbiota structure and *L. pneumophila* numbers with time. SWHs were equipped with copper or PEX pipe sections, dosed in triplicate with AOC from 0 to 700 μg/L, and subject to an incrementally increased temperature regime. Effects of copper pipe were further explored in complementary experiments in which Cu^2+^ was dosed into SWHs. In addition to Illumina amplicon sequencing of 16S rRNA genes, *L. pneumophila*, *Vermamoeba vermiformis*, *and Acanthamoeba* spp. gene markers were monitored by quantitative polymerase chain reaction (qPCR)*.* Insights gained help understand how the microbiota composition is shaped, intentionally and unintentionally, in hot water plumbing systems.

## Methods

### Temperature experiment

#### Simulated water heaters (SWHs)

SWHs consisted of 125-mL glass bottles equipped with PEX or copper pipe sections (8 Φ½ in. × 1.25 cm), a 2-mm layer of glass beads (Φ1 mm), and polytetrafluoroethylene caps. SWHs were operated at 32 °C for 2.5 years prior to the present study. During the 2.5 years, SWHs were initially fed with synthesized water and/or Blacksburg tap water (water was treated the same way as described previously [35], unfiltered with 0.45-μm filters) to allow the development of mature and complex drinking water biofilms in SWHs. SWHs were also inoculated with approximately 2.03 × 10^5^ gene copies/mL *L. pneumophila* (ATCC 33152, 33733, 33734, 33823, a mixture of environmental isolates, to maximize the likelihood that *L. pneumophila* established in the systems), 2.08 × 10^3^ gene copies/mL *Acanthamoeba polyphaga* (ATCC 30871), and 1.46 × 10^3^ amoeba/mL *Vermamoeba vermiformis* (ATCC 50237) after the first 4 months of the 2.5 years. On day 0 of the present study (defined as 2.5 years after initial setup), SWHs were cross-inoculated during a routine water change by pooling 100 mL water from each, mixing, and returning 1 mL of the mixture to each SWH together with 100 mL freshly prepared influent (details below). Cross-inoculation was done in this manner to limit interruptions to delivery of fresh nutrients during normal water changes.

#### Water changes

Influent water was prepared from local Blacksburg, VA, water, which is usually chloraminated. Influent was prepared by breakpoint chlorinating cold tap water through incremental addition of chlorine while measuring total residual chlorine using the Hach reagent Powder Pillows (Loveland, CO), totaling 2–5 mg/L chlorine addition. As per breakpoint chlorination kinetics with chloraminated waters, residual chlorine level initially increased, then decreased and increased again at the breakpoint, when chlorine addition was stopped. Breakpoint chlorinated water was further prepared by heating it to 90 °C for 10 min, passing it through a biologically active granular activated carbon (GAC) filter (Undercounter Standard Filter Unit, Model GX1S01R, General Electric, Fairfield, CT), filtering with a 0.45-μm polyvinylidene fluoride (PVDF) filter to remove colloidal carbon particles and most microbes to lower background carbon level and to reduce seasonal variations in influent water microbiota, and adjusting the pH to 7.5 ± 0.1. Three times a week, 100 of the 120 mL total volume was gently decanted (i.e., without mixing) and replaced with freshly prepared influent water. Water changes and sample collection were conducted using aseptic technique.

Five levels of influent AOC were investigated by dosing influent water with an acetate and glucose mixture (0, 5, 30, 150, and 700 μg C/L). While organic carbon in drinking water is typically complex in nature and not directly assimilable, as it is largely derived from natural organic matter and plumbing components, acetate and glucose were selected because they are highly assimilable by a wide variety of bacteria, common intermediates of degradation of more complex organic carbon forms, and commonly employed in assimilable organic carbon assays [36]. Triplicate SWHs represented each pipe material and AOC level.

#### Temperature experiment

Temperature was elevated in a stepwise fashion (2.5–4 °C every 5–7 weeks) in all SWHs, except for an extra temperature control set for each pipe material maintained at 32 °C and receiving 700 μg/L AOC. Samples were collected during regular water changes at the end of each temperature period, specifically, on days 1, 27, 58, 106, 141, 183, 224, 267, and 316, with temperatures of 32, 32, 32, 34.5, 37, 41, 45, 49, 53 °C, respectively.

Temperature was maintained with a New Brunswick Scientific Classic Series C24 Incubator Shaker (Edison, NJ, USA). Upon malfunction on days 274 and 277, SWHs were transferred to a New Brunswick Scientific Innova 43 Incubator Shaker (Edison, NJ, USA) for the remainder of the experiment. During malfunction, the incubator reached 60 °C for no more than 24 h.

#### Cu^2+^ dosing experiment

Six new SWHs were established to investigate the impact of copper ion (Cu^2+^) dosing. These new glass bottles were amended with PEX pipe sections (8 Φ ½ in. × 1.2 cm) and incubated at 32 °C. Water was prepared and changed as described above (no AOC dosed) three times per week. In week one (3 times change), influent was spiked with 34 mL pooled effluent from the temperature experiment SWHs. Starting from day 22, influent pH was adjusted from 7.5 to 7.0. Soluble Cu^2+^ was dosed into three SWHs at increasing levels (0, 5, 30, 150, and 1000 μg/L) using a stock 0.0014 M CuSO_4_ solution with 14 days between every increase, while the other three SWHs served as controls with no Cu^2+^ dosing. This experiment was run for a total of 90 days.

#### Copper ions

Copper ions (Cu^2+^) were measured using inductively coupled plasma mass spectrometry (ICP-MS). Ten milliliter from each SWH were acidified by adding 2% nitric acid by mass prior to analysis. Soluble copper was operationally defined by that passing through a 0.45-μm pore size filter.

#### Total organic carbon

Total organic carbon (TOC) was measured with a Sievers 5310 C Laboratory TOC-MS Analyzer using the Data Pro 5310 C Computer Program. Samples were analyzed using 30 mL from each SWH or by pooling 10 mL from each triplicate SWH. Samples were acidified with phosphoric acid and sparged with N_2_ gas to purge inorganic carbon prior to analysis.

#### DNA extraction

One hundred milliliter of SWH water was filtered onto sterile 0.22-μm pore size mixed cellulose ester filters (Millipore, Billerica, MA, USA). DNA was extracted using the FastDNA® SPIN Kit (MP Biomedicals, Solon, OH, USA) according to the manufacturer’s instructions.

#### Quantitative polymerase chain reaction

The quantitative polymerase chain reaction (qPCR) method was applied to quantify the macrophage infectivity potentiator (*mip*) gene specific to *L. pneumophila* [37], the 18S rRNA gene of *V. vermiformis* [38], the 18S rRNA gene of *Acanthamoeba* [39], and bacterial 16S rRNA genes [40] using protocols previously optimized for drinking water samples [41]. Q-PCR was carried out using a CFX96™ Real Time system (Bio-Rad, Hercules, CA, USA), including a calibration curve of seven to eight points and negative controls in each run.

#### Culturing

Culturing for detection of *L. pneumophila* was conducted by heating 100 μL water samples to 50 °C for 30 min and directly plating onto buffered charcoal yeast extract (BCYE) agar according to published methods [41, 42].

#### Illumina 16S rRNA gene amplicon sequencing

Illumina amplicon sequencing of bacterial 16S rRNA genes was applied to triplicate SWHs corresponding to both pipe materials, three levels of dosed AOC (0, 30, and 700 μg/L), and six temperature levels (32, 37, 41, 45, 49, and 53 °C), along with corresponding constant temperature controls sampled on the same days (days 58, 141, 183, 224, 267, and 316). 16S rRNA genes were amplified with barcoded primers targeting the V4 region according to the Earth Microbiome Project Illumina Amplification Protocol [43], followed by sequencing with an Illumina MiSeq Sequencer (2 × 250 bp) at the Virginia Bioinformatics Institute (Blacksburg, VA, USA). Sequences were deposited to the European Nucleotide Archive under project accession number PRJEB11665.

#### Data analysis

Microbial numbers (gene copies/mL water) were log-transformed [log_10_(x + 1)], with *L. pneumophila* numbers below detection treated as zero. *T* tests and correlation analyses were performed in JMP Pro 12 (Cary, NC, USA). 16S rRNA gene sequences were analyzed in mothur (1.36.0). Sequences with an average similarity ≥ 97% were assigned an operational taxonomic unit (OTU). Each sample was rarefied to 12,430 sequences for comparison. Alpha and beta diversities were determined as mean values of 1000 random rarefactions. Bray-Curtis similarity matrix and jclass matrix were applied to analysis of similarity (ANOSIM) in mothur, non-metric multi-dimensional scaling (NMDS) in Primer-E 6.0 (Plymouth, UK), and Adonis in R. Adonis analysis served to quantify the relative influences of temperature, pipe material, and dosed AOC level in shaping the hot water microbiota composition. “Persistent” OTUs were defined as being detected at all temperatures for any AOC level after rarifying to 12,430 sequences/sample. “Enriched” OTUs were those OTUs firstly identified by an indicator in mothur and then selected either having an indicator value > 80 or a relative abundance > 1%.

## Results

### Total bacterial numbers

AOC was amended to the SWHs as glucose and acetate and directly measured as TOC in the SWH influent and effluent at two time points, corresponding to the 37 and 53 °C experimental conditions. Influent TOC (TOC_in_) was confirmed to be proportional to the dosed AOC (*ρ* = 0.89–0.99) (Additional file 1: Fig. S1A). ΔTOC (ΔTOC = TOC_in_−TOC_out_) was calculated to represent reduction in organic carbon due to biodegradation and TOC generated (e.g., via autotrophy or leaching from pipes). At 37 °C, ΔTOC was positive (i.e., highly amenable to biodegradation) and correlated with dosed AOC for both pipe materials (Pearson correlation coefficient *ρ* = 0.87–0.88) (Additional file 1: Fig. S1B). At 53 °C, ΔTOC in SWHs with copper pipe remained low, regardless of the level of dosed AOC, suggesting that biodegradation was somewhat inhibited. ΔTOC values in SWHs with PEX pipe were consistently negative (TOC_out_ > TOC_in_), indicating leaching of organic carbon from PEX pipe. Under conditions amenable to biodegradation (37 °C with both pipe materials and 53 °C with copper), ΔTOC was strongly correlated with total bacterial numbers (measured as 16S rRNA gene copy numbers) (*ρ* = 0.87, *p* < 0.0001) (Additional file 1: Fig. S2), consistent with microbial activity consuming TOC.

**Fig. 1:**
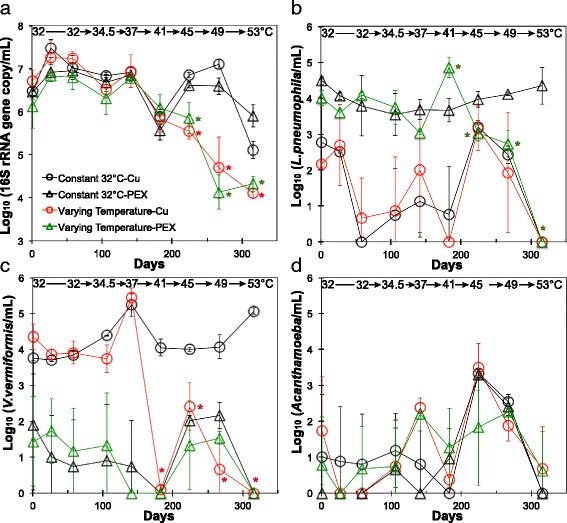
Effect of temperature and pipe material on gene copy numbers of total bacteria (**a**), *L. pneumophila* (**b**), *V. vermiformis* (**c**), and *Acanthamoeba* (**d**) in SWHs dosed with 700 μg/L AOC in the influent. All microbial numbers are log_10_-transformed gene copies/mL. SWHs with copper (circle) or PEX (triangle) pipe section were incubated constantly at 32 °C (black) or at increasing temperatures from 32 to 53 °C (red for copper, green for PEX) from day 1 to day 316. Error bars are standard deviations of triplicate SWHs

The effect of temperature was evaluated by comparing SWHs supplemented with 700 μg/L AOC and incubated at sequentially increasing temperatures versus the control SWHs maintained at 32 °C and also supplemented with 700 μg/L AOC. Total bacterial numbers in bulk water decreased significantly when temperatures are ≥ 45 °C (*p* < 0.05), while there was no apparent effect of pipe materials on total bacterial numbers at any temperature (Fig. 1a).

**Fig. 2:**
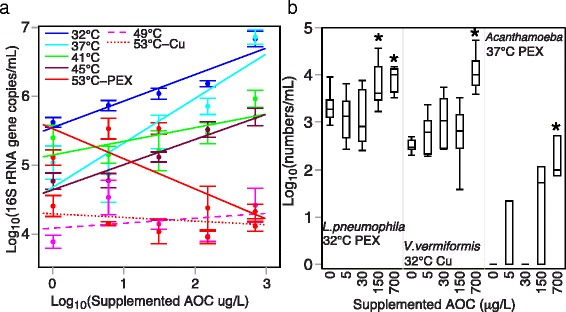
Effect of dosed assimilable organic carbon (AOC) on gene copy numbers. **a** Correlations between the numbers of total bacteria and dosed AOC level. Linear regression lines are color-coded according to temperature as indicated. Means and standard errors from replicated SWHs are shown (*n* = 6 for combined pipe material data at 32–49 °C, *n* = 3 for 53 °C, with each pipe material plotted individually). **b** Distribution of gene copy numbers of specific microbes of interest under temperature/pipe conditions where a significant effect of AOC supplementation was noted. For each boxplot, *n* = 9 for 32 °C and *n* = 3 for 37 °C. Asterisk indicates significant difference compared to lower AOC levels according to post hoc ANOVA test

An effect of dosed AOC on total bacterial numbers was observed at lower temperatures (Fig. 2a), with positive correlations noted from 32 to 45 °C (Pearson correlation *ρ* = 0.39–0.81, *p* < 0.05). No correlations were observed between dosed AOC and bacterial numbers at 49 °C (either pipe material) or 53 °C (copper pipe), while a negative correlation was observed for PEX pipe at 53 °C (*ρ* = − 0.66, *p* < 0.01).

### *L. pneumophila* and amoebae gene markers

Both pipe material and temperature significantly affected *L. pneumophila* and *V. vermiformis* gene copy numbers (hereafter “numbers”) (Fig. 2a). Positive correlations between individual microorganisms and supplemented AOC were only occasionally found (Fig. 2b), primarily driven by the highest dose(s) of AOC.

The effect of pipe material was best illustrated in the first three samplings, before increasing the temperature, and the control SWHs at 32 °C (Fig. 1). SWHs with PEX pipe had consistently higher numbers (2.5 log_10_) of *L. pneumophila* and lower numbers (3.1 log_10_) of *V. vermiformis* compared to SWHs with copper pipe.

**Fig. 3:**
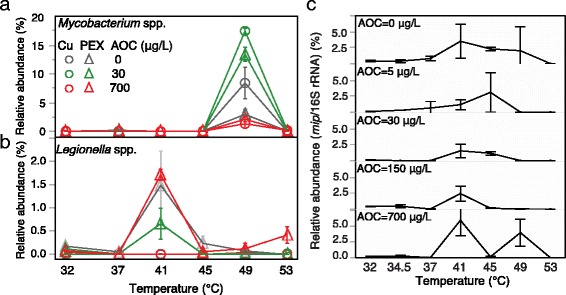
Effect of various factors on relative abundances of potential opportunistic pathogens. Changes in the relative abundance of **a** *Mycobacterium* spp. and **b** *Legionella* spp. with temperature based on Illumina sequencing. **c** Relative abundance of *L. pneumophila* based on qPCR (*mip* gene copies/16S rRNA gene copies) for PEX condition only. Water supplemented with 0–700 μg/L AOC were fed to SWHs and incubated at increasing temperatures (32–53 °C). Circles and triangles represent SWHs with copper and PEX pipe sections, respectively. Error bars represent standard errors of triplicate SWHs

Temperature influenced *L. pneumophila* in SWHs with PEX (Fig. 1b), where numbers significantly increased and peaked at 41 °C, followed by a drop at 45 and 49 °C, and a further drop to below-detection at 53 °C (*p* < 0.05 in all cases relative to the 32 °C control). The relative abundance of *L. pneumophila* among total bacteria, estimated as the ratio of *mip* to 16S rRNA gene copy numbers, peaked between 41 and 49 °C (Fig. 3c). Similarly, temperature influenced *V. vermiformis* in SWHs with copper (Fig. 1c). *V. vermiformis* numbers decreased significantly to a level either below detection or 2-log_10_ less than control reactors when the temperature was ≥ 41 °C. When numbers were low, impacts of material on *Acanthamoeba* (Fig. 1d) or temperature on *L. pneumophila* (in copper SWHs) and *V. vermiformis* (in PEX SWHs) could not be discerned.

### Copper ion (Cu^2+^) dosing

**Fig. 4:**
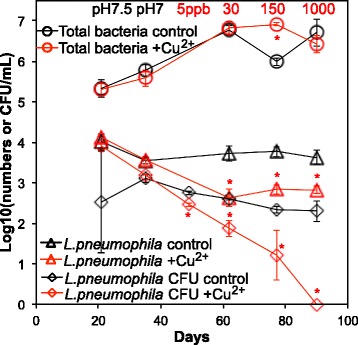
Effect of copper ion dosing on total bacteria and *L. pneumophila*. Total bacteria (16S rRNA gene copy numbers—circles) and *L. pneumophila* (*mip* gene copy numbers—triangles or CFU—diamonds) were measured in SWHs with PEX pipe at 32 °C. No AOC was supplemented. An increasing amount of Cu^2+^ was dosed over the course of the experiment, from 5 to 1000 ppb as shown in the top panel, to the SWHs indicated in red. Standard errors were determined from triplicate SWHs. Asterisk indicates significant difference in comparison to the no Cu^2+^ control

In a separate set of SWHs, increasing Cu^2+^ dose from 5 μg/L up to 1000 μg/L with time showed no effect on total bacterial numbers, but resulted in a significant decrease in the *L. pneumophila* numbers, both in terms of gene copies and cultivable bacteria (CFU/mL) (Fig. 4). Cu^2+^ dosing higher than 30 μg/L did not further reduce *L. pneumophila* gene copy numbers, but did reduce the cultivable number of *L. pneumophila*. There was no observed effect of Cu^2+^ dosing on *Acanthamoeba* numbers (Additional file 1: Fig. S3).

### Microbiota composition

**Fig. 5:**
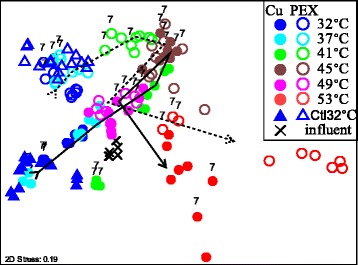
NMDS plot illustrating the overall dissimilarities among hot water microbiota. Circles represent SWHs with copper (solid) or PEX (open) pipe incubated at varying temperatures (blue, 32 °C; cyan, 37 °C; green, 41 °C; brown, 45 °C; magenta, 49 °C; red, 53 °C) in comparison the control set maintained at 32 °C (triangles, Ctl32°C). Cross symbols represent influent water (after GAC filtration) at the time of 37 °C incubation. Labels of “7” above a symbol indicate 700 μg/L dosed AOC, while non-labeled conditions represent 0 and 30 μg/L dosed AOC. The trajectory lines (solid—copper; dashed—PEX) indicate the trend of microbiota changing with increasing temperatures

Each sample was rarefied to 12,430 sequences, and an NMDS plot was generated based on pair-wise Bray-Curtis dissimilarities (Fig. 5). The cold tap water influent samples consistently clustered separately from the SWH samples, illustrating a distinct hot water microbiota. When the temperature was ≤ 41 °C, pipe material was the dominant factor shaping the composition of the microbiota. At 45 and 49 °C, the composition of the microbial communities converged, regardless of the pipe material, but at 53 °C the effects of PEX and copper were again apparent with greater variation among replicates. Samples from SWHs maintained constantly at 32 °C through time remained clustered, providing confirmation that the observed shifts in microbiota composition were driven by temperature rather than temporal variation.

According to Adonis analysis (Table 1), temperature was the primary driver of the majority of variation (43%) in the microbiota composition, while pipe material and AOC were associated with a significant, but smaller impact (explaining 7 and 4% of the variation, respectively). The effect of pipe material surpassed that of dosed AOC level at temperatures ≤ 45 °C by other analyses as well (ANOSIM, *p* < 0.01, Additional file 1: Table S1). The effect of dosed AOC was observed primarily among comparisons within the same temperature/pipe material. The addition of 30 μg/L AOC was significant only at the two lowest temperatures (32 and 37 °C, *p* < 0.05). Dosing of 700 μg/L AOC resulted in a significant effect, with a distinct microbiota composition from that of lower AOC levels at each temperature (32–53 °C, *p* < 0.05) with the same pipe material (Additional file 1: Table S1).

**Table 1:** Relative impacts of the three factors and their interactions on hot water microbiota. Results shown as analysis of similarity (ANOSIM) global R statistics

Factors	Explained variations in microbiota	Overarching factor test ANOSIM
Temperature (T)	43%*	*R* = 0.63*
Pipe material (M)	7%*	*R* = 0.16*
AOC (C)	4%*	*R* = 0.002 (*p* > 0.05)
T × M	14%*	–
T × C	12%*	–
C × M	3%*	–
T × C × M	7%*	–
Residuals	11%	–

*Indicates *p* value < 0.001

**Fig. 6:**
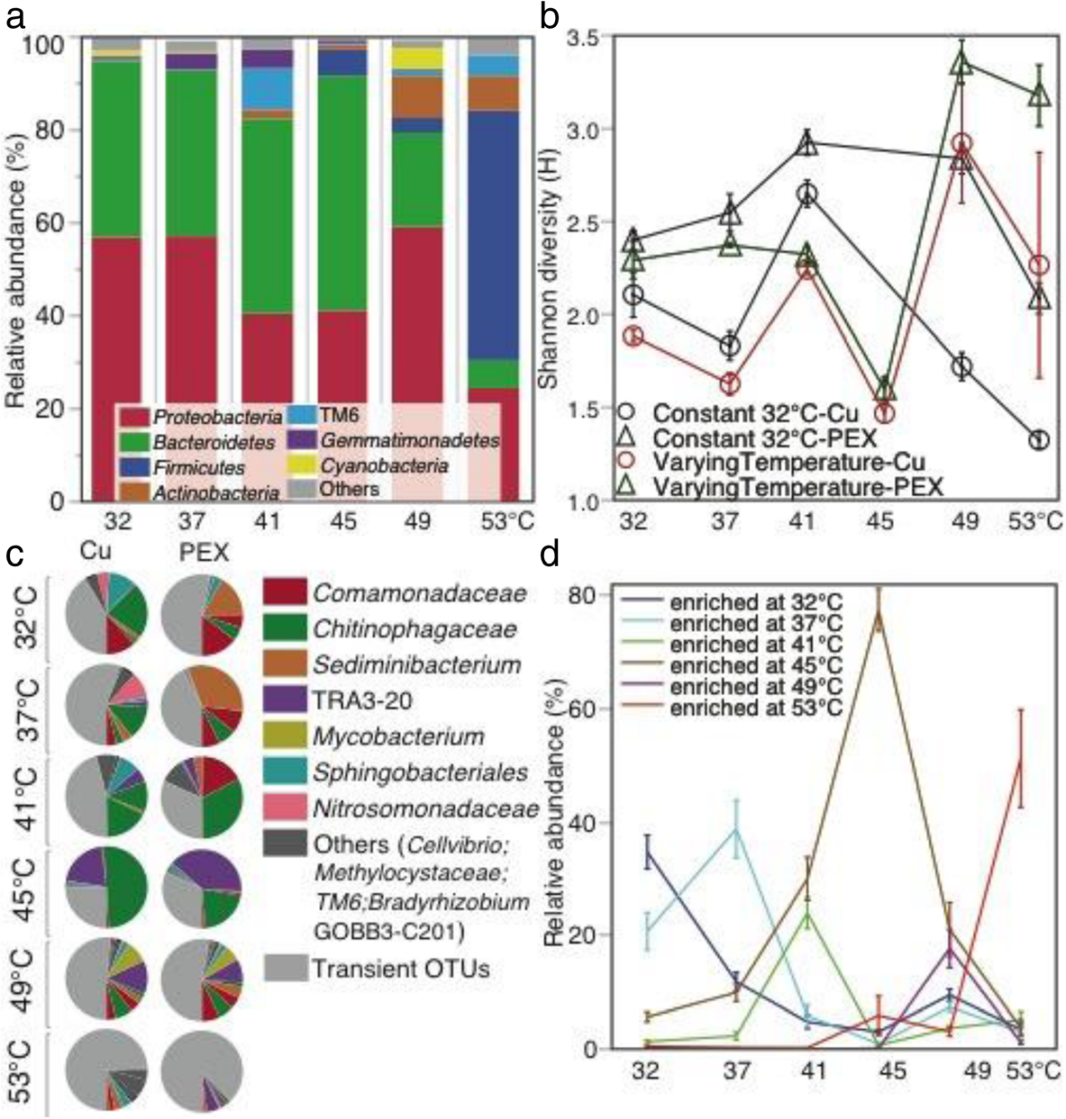
Effects of temperature and pipe material on microbiota composition and diversity. **a** Phylum composition, (**b**) Shannon diversity, (**c**) 14 persistent OTUs at all temperatures for both pipe materials, and (**d**) OTUs selectively enriched with temperature. OTUs being undetected in any temperature or pipe material are grouped as “Transient OTUs”. Standard errors were determined from triplicate SWHs (**b** and **d** panels). Each plotted line in panel **d** represents three to five OTUs enriched at each temperature (see Additional file 1: Table S2 for details)

The two most dominant phyla switched from *Proteobacteria* (57%) and *Bacteroidetes* (37%) at 32 °C to *Firmicutes* (54%) and *Proteobacteria* (25%) at 53 °C (Fig. 6a). *Actinobacteria* populations were also enriched at higher temperatures (49 and 53 °C).

### Microbial diversity

Shannon diversity decreased as temperature increased from 41 to 45 °C, but then significantly increased (1.2–1.7 times) at 49 and 53 °C, regardless of pipe material (compared to temporal changes observed in the control SWHs, Fig. 6b). Together with increased diversity (Shannon index) at 49 and 53 °C, there was more evenness (1.3–1.8 times for both copper and PEX) and lower richness (0.4–0.5 times for PEX only). SWHs with PEX usually had higher Shannon diversity than copper. The effects of dosed AOC level on Shannon diversity varied with temperature (Additional file 1: Fig. S4). The highest AOC level (700 μg/L) resulted in a lower Shannon index, with few exceptions (copper and PEX at 41–45 °C, PEX at 53 °C).

### OTUs persisting across temperatures and pipe materials

Some OTUs persisted from warm (32 °C) to hot (53 °C) conditions, indicating their resistance or tolerance to this substantial temperature shift. A total of 14 OTUs were detected across all temperatures in both copper and PEX (Fig. 6c). Although their relative abundances shifted with temperature and pipe material, together, these persistent OTUs accounted for ~ 50% of the microbiota at all temperatures except 53 °C (< 20% at 53 °C).

### OTUs selectively enriched at various temperatures

Some OTUs were enriched mainly at a specific temperature. Each set of 4–7 OTUs was identified to have significantly higher abundance at one temperature than all other temperatures, across both pipe materials and all AOC levels (Fig. 6d, Additional file 1: Table S2). The selectively enriched OTUs at 41, 49, or 53 °C were highly specific to temperature, displaying high abundance (18–78%) at the corresponding temperature, but becoming “rare” (total abundance < 5%) at all other temperatures.

### OTUs selectively enriched by different pipe materials

**Fig. 7:**
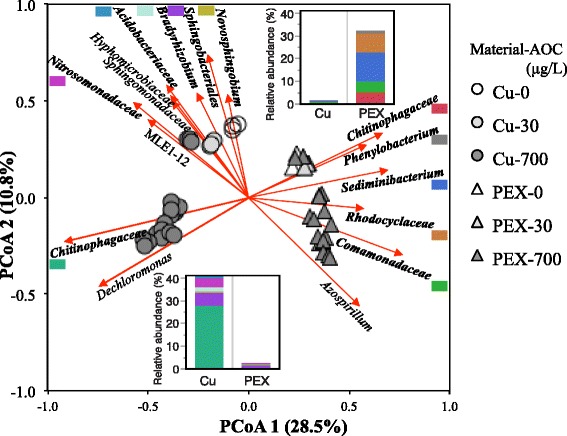
Impact of pipe material on hot water microbiota illustrated using PCoA plot of SWHs with copper (circles) or PEX (triangles) pipe sections supplemented with 0 (white), 20 (light gray), or 700 (dark gray) microgram per liter AOC and incubated at 32 °C. The most abundant OTUs significantly correlated with both axes are shown as the arrows and labeled with their best-known taxonomy identification. In bold are OTUs significantly enriched in one pipe material at all lower temperatures (32–41 °C). Embedded figures show the relative abundance of copper (lower) and PEX (upper) enriched OTUs in SWHs at 32 °C

Based on PCoA analysis of all samples at 32 °C (Fig. 7), over a dozen OTUs were identified as enriched by either PEX (positive correlation with PCoA1 axis) or copper (negative correlation with PCoA1 axis). Additionally, five or six of these OTUs were enriched in PEX or copper across all lower temperatures (32–41 °C), accounting for 30–40% of the community in the corresponding pipe material and < 5% in the other pipe material.

### Genera containing pathogens

Several genera were of particular interest because they potentially contain OPs. Temperature was the most influential factor to the relative abundances of *Mycobacteria*, *Pseudomonas* and *Stenotrophomonas*, peaking at 49, 53, and 53 °C, respectively, regardless of pipe material (Fig. 3a, Additional file 1: Fig. S5). Dosed AOC level up to 30 μg/L was associated with an increase in the relative abundance of *Mycobacteria*. Among the four OP-containing genera tracked in this study, only the relative abundance of *Legionella* was significantly influenced by pipe material (Fig. 3b), remaining low in SWHs with copper (< 0.2%), but peaking at 41 °C with a relative abundance of >1.5% of the total microbial composition in the PEX pipe condition.

### Variance among replicates

Variance in microbiota composition among SWH replicates (estimated as pairwise Bray-Curtis dissimilarities) increased markedly at 49 and 53 °C in SWHs with copper pipe, but remained relatively stable in SWHs with PEX over the range of temperatures (Additional file 1: Fig. S6). Dissimilarities among replicates were higher in PEX (~ 30%) than copper (~ 12%) among SWHs at lower temperatures (≦45 °C), with most dissimilarity among replicates attributed to the 700 μg/L AOC condition.

### Comparison of microbiota and qPCR profiling

**Fig. 8:**
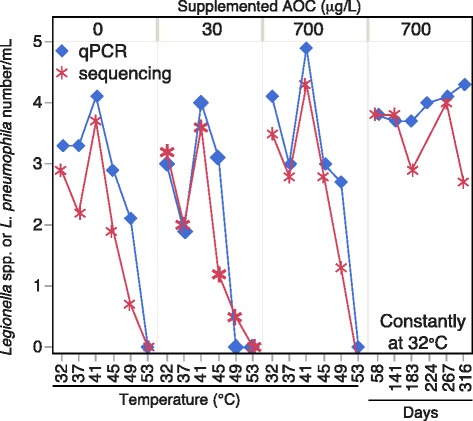
Absolute number of *L. pneumophila* quantified by qPCR in comparison to the number of *Legionella* spp. determined by Illumina sequencing. To determine absolute number of *Legionella spp.* using Illumina sequencing information, the relative abundance (%) of OTUs belonging to the genera *Legionella spp.* was multiplied with the number of 16S gene copies quantified qPCR

Absolute abundance of *Legionella* spp. could be roughly calculated by multiplying the relative abundance of the genus from Illumina sequencing data with the absolute number of 16S rRNA gene copies determined with qPCR. Such methods have been employed previously [34, 44]. When comparing results from this method with the absolute number of *L. pneumophila* determined with qPCR, highly similar trends were produced (Fig. 8), with the absolute numbers of *L. pneumophila* and *Legionella* spp. both peaking at 41 °C.

We further compared microbiota and qPCR analyses by correlating the relative abundances of *L. pneumophila* calculated from qPCR with the relative abundances of the 100 most dominant OTUs identified by the amplicon sequencing. The strongest positive correlation with *L. pneumophila* numbers was a *Legionella* OTU (OTU69) (*ρ* = 0.47)*.* The second highest correlation was with an OTU with a high sequence similarity to *Methylophilus* (*ρ* = 0.37), which is a known endosymbiont of *Acanthamoeba* [45].

## Discussion

Temperature, material, and AOC all affected hot water microbial composition and *L. pneumophila* occurrence. Here, we systematically discuss the relative importance of each factor and interactive effects among them.

### Temperature

While temperature control is an established method of preventing OP regrowth [10], recommendations for water heater settings vary due to conflicting priorities and goals [11–14]. In this study, a temperature of 53 °C uniformly reduced *L. pneumophila* and *V. vermiformis* (a key host for *L. pneumophila*) to below detection, regardless of AOC or pipe material conditions (Fig. 1b, c). This supports the concept of temperature as a diagnostic of risk of OP colonization [10]. It also suggests that 55 °C, a temperature considerably lower than the standard set point 60 °C, can technically be effective if achieved homogeneously in the system, as was the case in these SWHs. However, water temperature in realistic water heaters and hot water distribution pipes is typically lower than the set point due to cooling in distal pipes or mixing with cold water [8, 46]. In a recent pilot-scale simulation, *L. pneumophila* remained detectable at the tap and in the recirculating line even when the water heater was set at 58 °C [8].

Based on OP-containing genera identified by amplicon sequencing, mid-range temperatures (41–49 °C) could be more problematic than the assumed “worst-case scenario” of 32 °C. For example, *Mycobacterium* spp., while consistently detected at all temperatures in both pipe materials, peaked at 49 °C (Fig. 3a). *L. pneumophila* numbers peaked in PEX reactors when maintained at 41 °C (Fig. 1b), matching a spike in calculated relative abundance and both relative and calculated absolute abundance of *Legionella* spp. based on amplicon sequencing (Figs. 3 and 8). The peak concentrations of *L. pneumophila* in this study (5.2 log_10_ *mip* gene copies/mL) may even surpass the critical threshold for *L. pneumophila* infection of 10^7^CFU/L, which was identified in a quantitative microbial risk assessment model [47].

While temperature was the most influential factor inducing differences in microbiota structure, there were 14 persistent OTUs that dominated at most temperatures for both materials (Fig. 6c). These OTUs include some implicated in regrowth during stagnation (*Comamonadaceae*, *Bradyrhizobium*) [48], some commonly identified in drinking water biofilms (*Mycobacterium* and *Sphingobacteriales*) [34], some likely involved in nitrogen cycling (*Nitrosomonadaceae*) [49] and some potential methanotrophs known to degrade chlorine byproducts (*Methylocystaceae*) [50]. The former two are likely related to the imposed hydraulic cycle of 2–3 days between water changes and constant seeding from mature SWH biofilms, a phenomenon previously observed in other systems [16]. The latter two may indicate that the breakdown of disinfection byproducts continues to influence SWHs, even though the influent water was breakpoint chlorinated.

The microbiota shift at 53 °C was mostly attributed to *Firmicutes*. While *Firmicutes* were previously associated with adoption of a new chloramine disinfection system in a hospital [51], in this study, it is their thermophilic nature that likely provided them a selective advantage [52]. *Actinobacteria* were also enriched at this temperature, consistent with the selective advantage of high GC content and Gram-positive characteristics at high temperature.

Interestingly, the community changes occurring at each temperature increase could be largely accounted for by just a few OTUs that were enriched at individual temperatures (Fig. 6d). These OTUs are particularly interesting at 41 °C, when *L. pneumophila* peaked in PEX reactors and the communities with both materials began to converge. *Novosphingobium*, an aromatic carbon degrader [53], and *Caulobacter* were favored. *Caulobacter* was previously associated with shower hose biofilms that formed under similar conditions (41 °C with 24-h stagnation) and consistently contained *Legionella* [34]. Together with the previously mentioned *Methylophilus*, these OTUs may be indicators for *Legionella* issues.

This study clearly indicates that temperature will induce shifts in hot water microbial structure, even with differences as little as 4 °C. While previous studies have shown wide-scale differences between hot and cold water microbiota [16], to the authors’ knowledge, this is the first study to examine such fine-scale adaptation to temperature under conditions representative of building plumbing. The fine-scale temperature adjustment approach selected here allowed the microbial community to gradually adapt and helped to identify threshold temperatures likely to be effective for opportunistic pathogen control under various conditions. Such fine-scale adjustments may also be made in the real world in seeking to address concerns such as energy, scalding, or cost. It is important to note, however, that a dramatic “heat-shock” type temperature adjustment would likely have resulted in microbial community structures distinct from those observed here. While incremental shifts may allow bacteria to build up resistance to higher temperature, sudden increase from 32 to 53 °C would trigger a heat-shock response [54].

### Pipe material

Copper pipe had no measureable effect on total bacterial numbers in our long-term aged pipe study (Fig. 1a). Although copper pipes may retard initial biofilm formation relative to plastic pipes [22], often, studies are conducted using a new pipe material, where the effects of carbon leaching from plastics and Cu^+2^ from copper pipes are greatest. This experiment was conducted with pipes that were experimentally operational and aged for > 3 years, greatly exceeding prior studies of 30 days [55], 1 year [16, 18], and 2 years [21]. Although a general toxicity of copper on total bacterial numbers was not observed, aged copper pipe exerted selective pressure that benefitted some microorganisms over others, as illustrated by the microbiota composition (Fig. 7) and response of specific OPs and organisms of interest.

There is some evidence that copper pipe can be beneficial for some free-living amoeba (FLA) hosts for *L. pneumophila* [56]*.* In this study, SWHs with aged copper pipes had higher concentrations of *V. vermiformis* than PEX reactors at low temperatures (Fig. 1b), while *Acanthamoeba* did not respond to Cu^+2^ dosing (Additional file 1: Fig. S3). Although co-occurrence of FLA and *L. pneumophila* has been reported [57], specific FLA and *L. pneumophila* may respond differently to stresses like temperature [58], potentially explaining the discrepancy in *L. pneumophila* versus *V. vermiformis* response in this study. Additionally, although potential copper pipe toxicity towards *Mycobacterium* was observed in a prior building survey [16], *Mycobacterium* was persistent on both materials in this study.

Below 45 °C, copper pipe consistently suppressed *L. pneumophila* numbers compared to PEX, even after 2.5 years of conditioning and operation of the present experiment for over a year (Fig. 1b). The Cu^+2^ dosing study made it possible to further examine the selective pressure. A concentration as low as 5–30 μg/L effectively inhibited *L. pneumophila* at 32 °C (Fig. 4), indicating that the lower *L. pneumophila* numbers in SWHs with aged copper pipe than PEX pipe at day 1 may be due to its sensitivity to Cu^+2^ released from copper pipe during the acclimation period.

At lower temperatures, there was a clear distinction in the microbiota structure associated with copper versus PEX (Fig. 7). The OTUs associated with PEX represented a variety of ecological niches consistent with the leaching of diverse organic carbon from the plastic [19, 23]. For example, *Phenylobacterium* has a specific metabolism associated with herbicides and complex carbon [59] and *Sediminibacterium* was previously associated with biofilms on PVC-P [34]. The wide range of carbon niches could also account for the higher Shannon diversity observed in PEX SWHs. Previously, a higher diversity was also observed in biofilms on plastic than on copper [56].

The OTUs specific to the copper condition were associated with adaptive metabolism and resistance to harsh environments, likely reflecting the toxicity of the copper pipes and lowered carbon availability compared to the PEX condition. For example, some are known for tolerance to acidic conditions (*Acidobacteriaceae*) [60] and preference for low organic carbon levels (*Hyphomicrobiaceae*) [61]. *Pseudomonas* was also persistent in Cu SWHs, and the opportunistic pathogen, *P. aeruginosa*, has previously demonstrated copper resistance [62]*.* At higher temperatures, the distinction between materials became less obvious.

Prior studies have yielded conflicting conclusions with respect to the relative effects of copper versus plastic (PEX, PVC, C-PVC) pipes on levels of *L. pneumophila* and other organisms [16, 18, 21, 22, 55, 63, 64]. However, since temperature can alter the water chemistry (e.g., pH) and overall tendency towards dissolution of copper ions from pipes [65], it is critical to consider both water chemistry and physiochemical parameters when determining the effectiveness of copper pipe in controlling *L. pneumophila*. We hypothesize that the influence of pipe material is highly dependent on water temperature, which governs both leaching of organic carbon from plastics and copper dissolution chemistry.

In this study, higher temperature decreased the dissolution of total copper from the pipe (Additional file 1: Fig. S7), and further, this copper also likely had decreased solubility [66]. Thus, as one selective pressure was increased (temperature), another selective pressure was relieved (copper), perhaps accounting for the converging communities and similar response of *L. pneumophila* between pipe materials at these temperatures. SWHs with aged copper pipe at high temperatures had a noticeable increase in microbiota dissimilarity among replicates (Additional file 1: Fig. S6), possibly because new niches opened up by reduced copper pressure were quickly occupied by different organisms among the replicates.

At 53 °C, there was strong evidence of organic carbon leaching from the PEX pipes. The negative ΔTOC in PEX reactors at higher temperature indicated that leaching of organic carbon from the pipe exceeded the organic carbon consumed by microbes. Although the pipes were aged and carbon leaching tends to diminish over time, higher temperatures can still greatly increase the amount of TOC leaching from pipes [19]. There was also a strong shift in the microbiota structure at 53 °C in PEX reactors, especially when dosed AOC was low (0, 30 μg/L) (Additional file 1: Fig. S8). The communities with little dosed AOC (0, 30 μg/L) likely adapted to utilize the newly dominant PEX-derived carbon source, while those with higher dosed AOC (700 μg/L) could continue the previous metabolic patterns utilizing dosed AOC.

### AOC

AOC has been extensively studied in water distribution main systems (from treatment plant or source to consumers), with reduction below 100 or 10 μg/L AOC associated with effective control of regrowth, depending on other limiting factors [30–32]. However, these limits have not been validated for controlling regrowth within the hot water plumbing environment, where AOC can also be generated from pipes, especially when stagnation events greater than 6 h are common [34, 46]. In this experiment, the incoming carbon concentration was first minimized via biologically active GAC filtration, in order to reduce the background TOC/AOC and exclude potential effects of seasonal AOC variations.

Increasing AOC levels resulted in more bacteria at temperatures ≤ 45 °C, consistent with consumption of TOC and incorporation into biomass (Fig. 2a, Additional file 1: Figs. S1 and S2). However, in PEX reactors at 53 °C, there was a negative correlation between dosed AOC and 16S rRNA genes (Fig. 2a). With temperature-dependent leaching from pipes, local carbon production diminished the importance of the influent water, as has been previously observed with plastic pipes [34, 67].

Although other studies have found correlations between TOC and *L. pneumophila* in flowing hot [27] and cold water systems [68], in this study, only the highest levels of supplemented AOC corresponded to an increase in *L. pneumophila* or other organisms of interest, while high levels even seemed to suppress *Mycobacteria*. Thus, there may be a threshold at which AOC control is no longer effective for specific OPs, as suggested by Williams et al. [35]. Carbon production within the building (e.g., plastics leaching carbon) and specific preferences of potential pathogens may change the community response to increased carbon. For example, some organisms, like *Mycobacteria* and *P. aeruginosa*, may prefer a lower range of AOC because they can out-compete others for carbon in oligotrophic stagnant environments, as has been previously observed [34, 69]. Regardless, *L. pneumophila* and FLA are not directly dependent on carbon available in the water, especially under warm stagnant conditions, but rather derive largely secondary influences of carbon and broader microbiota as an intermediary [70, 71].

Finally, the low Shannon diversity and high dissimilarity among replicate SWHs in the 700 μg/L AOC condition may be related to the semi-batch nature of the reactors. A high concentration of easily assimilable organic carbon may select for fast-growing organisms that out-compete others for secondarily limiting nutrients (nitrogen, phosphorus), ultimately limiting diversity. Which bacteria dominate in semi-batch growth reactor can vary considerably among replicates. A notable exception to this trend occurred in PEX reactors at 53 °C, when carbon leaching introduced new carbon sources that were better utilized in the low carbon addition conditions.

## Conclusions

This study brings to light the importance of interactive effects of temperature, pipe material, and AOC in shaping the microbiota composition and controlling OPs in building plumbing. While temperature was the strongest factor, increasing temperature did not linearly reduce concentrations of OPs or amoeba hosts, which peaked at temperatures 41–49 °C. *L. pneumophila* and the two amoebae were reduced to below detection when the temperature was consistently maintained at 53 °C. Temperature also mediated the leaching behavior of pipes, reducing the selectively inhibitive effect of copper pipes and increasing TOC release from plastic pipes. AOC correlated with total bacteria, but not with *L. pneumophila*.

## Additional file

Additional file 1:
**Fig. S1**. Correlations between (A) influent TOC and (B) ΔTOC (TOCin-out) with supplemented AOC at 37 and 53 °C. **Fig. S2**. Linear correlation between total bacterial numbers and ΔTOC (TOCin-TOCout) in SWHs at 37 °C (copper and PEX) and 53 °C (copper). **Fig. S3**. Impact of copper dosing on the number of *Acanthamoeba* spp. in SWHs with PEX pipe at 32 °C. **Fig. S4**. Effect of supplemented AOC on Shannon diversity in SWHs with copper (red) or PEX (blue) pipe at each temperature (32–53 °C). **Fig. S5**. Changes of relative abundance of (A) *Pseudomonas* spp. and (B) *Stenotrophomonas* spp. with temperature according to identification based on 16S rRNA gene amplicon sequencing. **Fig. S6**. Bray-Curtis dissimilarities among replicate SWHs. **Fig. S7**. Dissolved copper ions in SWH bulk water. **Fig. S8**. Effect of supplemented AOC and pipe material on phylum composition in hot water at each temperature (32–53 °C). **Table S1**. Impacts of pipe material and AOC on microbial composition at each temperature (shown as ANOSIM global R statistics). **Table S2**. Enriched OTUs at each temperature. (DOCX 1453 kb)
