# Supplementary material for: Interactive effects of temperature, organic carbon, and pipe material on microbiota composition and Legionella pneumophila in hot water plumbing systems
Source: Microbiome. 2017 Oct 4;5:130. doi: 10.1186/s40168-017-0348-5 (PMC5628487; doi:10.1186/s40168-017-0348-5)
Supplement: Additional file 1: — Fig. S1. Correlations between (A) influent TOC and (B) ΔTOC (TOCin-out) with supplemented AOC at 37 and 53 °C. Fig. S2. Linear correlation between total bacterial numbers and ΔTOC (TOCin-TOCout) in SWHs at 37 °C (copper and PEX) and 53 °C (copper). Fig. S3. Impact of copper dosing on the number of Acanthamoeba spp. in SWHs with PEX pipe at 32 °C. Fig. S4. Effect of supplemented AOC on Shannon diversity in SWHs with copper (red) or PEX (blue) pipe at each temperature (32–53 °C). Fig. S5. Changes of relative abundance of (A) Pseudomonas spp. and (B) Stenotrophomonas spp. with temperature according to identification based on 16S rRNA gene amplicon sequencing. Fig. S6. Bray-Curtis dissimilarities among replicate SWHs. Fig. S7. Dissolved copper ions in SWH bulk water. Fig. S8. Effect of supplemented AOC and pipe material on phylum composition in hot water at each temperature (32–53 °C). Table S1. Impacts of pipe material and AOC on microbial composition at each temperature (shown as ANOSIM global R statistics). Table S2. Enriched OTUs at each temperature. (DOCX 1453 kb) [file 40168_2017_348_MOESM1_ESM.docx]

**Supplementary Information for:**

**Interactive Effects of Temperature, Organic Carbon, and Pipe Material on Microbiota Composition and *Legionella pneumophila* in Hot Water Plumbing Systems**

Caitlin R. Proctor^§,^^[[1]](#footnote-1),^ Dongjuan Dai^§^, Marc A. Edwards, Amy Pruden^*^

Via Department of Civil and Environmental Engineering, Virginia Tech, Blacksburg, Virginia 24061, United States

^§^ These authors contributed equally to this work.

Current address: EAWAG, Swiss Federal Institute of Aquatic Science and Technology, Überlandstr. 133, CH-8600, Duebendorf, Switzerland

^*^Corresponding author: apruden@vt.edu; (phone) (540)231-3980

Remaining authors’ emails: Caitlin.Proctor@eawag.ch, ddai@vt.edu, edwardsm@vt.edu

**Figure S1.** Correlations between A) influent TOC and B) ΔTOC (TOC_in-out_) with supplemented AOC at 37 °C and 53 °C.

**Figure S2.** Linear correlation between total bacterial numbers and ΔTOC (TOC_in_-TOC_out_) in SWHs at 37 °C (copper and PEX) and 53 °C (copper).

**Figure S3.** Impact of copper dosing on the number of *Acanthamoeba* spp. in SWHs with PEX pipe at 32 °C.

**Figure S4.** Effect of supplemented AOC on Shannon diversity in SWHs with copper (red) or PEX (blue) pipe at each temperature (32-53 °C)

*Pseudomonas* spp.

*Stenotrophomonas* spp.

**Figure S5.** Changes of relative abundance of A) *Pseudomonas* spp. and B) *Stenotrophomonas* spp. with temperature according to identification based on 16S rRNA gene amplicon sequencing.

**Figure S6.** Bray-Curtis dissimilarities among replicate SWHs.

**Figure S7.** Dissolved copper ions in SWH bulk water. Copper was measured with ICP analysis at three temperatures – 45 °C, 49 °C, and 53 °C. Concentration was also measured in 32 °C temperature control reactors at the same time. Measurements were made in both Copper and PEX reactors.

**Figure S8.** Effect of supplemented AOC and pipe material on phylum composition in hot water at each temperature (32-53 °C)

**Table S1.** Impacts of pipe material and AOC on microbial composition at each temperature (shown as ANOSIM global R statistics)

| Temperature (°C) | 32 | 37 | 41 | 45 | 49 | 53 |
| --- | --- | --- | --- | --- | --- | --- |
| Pipe material (over-arching AOC level)  Composition  Membership | **0.74^*^** | **0.80^**^** | **0.81^**^** | **0.65^**^** | 0.02 | 0.37^*^ |
|  | **0.99^**^** | **0.97^**^** | **0.83^**^** | 0.20^*^ | 0.00 | 0.18^*^ |
| Among AOC levels (within the same material) | **0*30**  ***700** | **0*30**  ***700** | 0-30  ***700** | 0-30  ***700** | 0*30  ***700** | 0-30  ***700** |

^*^ p< 0.05; ^**^ p<0.01; - not significant (p>0.05)

**Table S2.** Enriched OTUs at each temperature

| **OTUs enriched at 32°C** | **Taxonomy** |
| --- | --- |
| OTU000001 | *Sphingobacteriales* |
| OTU000017 | *Sphingobacteriales* |
| OTU000022 | *Rhodobacteraceae* |
| OTU000034 | *Azospira* |
| OTU000031 | *Sphingomonadaceae* |
| OTU000028 | *Rhodobacteraceae* |
| OTU000071 | *Comamonadaceae* |
| **OTUs enriched at 37°C** | **Taxonomy** |
| OTU000006 | *Sediminibacterium* |
| OTU000013 | wr0007 |
| OTU000007 | *Rhodocyclaceae* |
| OTU000023 | *Nitrosomonadaceae* |
| OTU000043 | *Phenylobacterium* |
| OTU000051 | *Sphingopyxis* |
| **OTUs enriched at 41°C** | **Taxonomy** |
| OTU000018 | *Novosphingobium* |
| OTU000040 | Candidate_division_TM6 |
| OTU000049 | *Caulobacter* |
| OTU000025 | *Ralstonia* |
| **OTUs enriched at 45°C** | **Taxonomy** |
| OTU000002 | *Chitinophagaceae* |
| OTU000010 | TRA3-20 |
| OTU000033 | *Chitinophagaceae* |
| OTU000014 | *Niabella* |
| **OTUs enriched at 49°C** | **Taxonomy** |
| OTU000011 | *Mycobacterium* |
| OTU000020 | *Sphingomonas* |
| OTU000038 | *Nevskia* |
| OTU000036 | *Porphyrobacter* |
| OTU000055 | MLE1-12 |
| **OTUs enriched at 53°C** | **Taxonomy** |
| OTU000003 | *Firmicutes* |
| OTU000047 | *Staphylococcus* |
| OTU000045 | GOBB3-C201 |
| OTU000065 | *Planctomycetaceae* |
| OTU000124 | *Bacillales* |

1. [↑](#footnote-ref-1)
